# Diversity of the Peruvian Andean maize (*Zea mays* L.) race *Cabanita*: Polyphenols, carotenoids, *in vitro* antioxidant capacity, and physical characteristics

**DOI:** 10.3389/fnut.2022.983208

**Published:** 2022-09-26

**Authors:** Iraida Sara Fuentes-Cardenas, Rody Cuba-Puma, Shaneri Marcilla-Truyenque, Huber Begazo-Gutiérrez, Gastón Zolla, Claudia Fuentealba, Kalidas Shetty, Lena Gálvez Ranilla

**Affiliations:** ^1^Laboratory of Research in Food Science, Universidad Catolica de Santa Maria, Arequipa, Perú; ^2^Estación Experimental Agraria Arequipa, Instituto Nacional de Innovación Agraria (INIA), Arequipa, Perú; ^3^Laboratorio de Fisiologia Molecular de Plantas, PIPS de Cereales y Granos Nativos, Facultad de Agronomia, Universidad Nacional Agraria La Molina, Lima, Perú; ^4^Escuela de Alimentos, Facultad de Ciencias Agronómicas y de los Alimentos, Pontificia Universidad Católica de Valparaíso, Valparaíso, Chile; ^5^Department of Plant Sciences, North Dakota State University, Fargo, ND, United States; ^6^Escuela Profesional de Ingeniería de Industria Alimentaria, Facultad de Ciencias e Ingenierías Biológicas y Químicas, Universidad Catolica de Santa Maria, Arequipa, Perú

**Keywords:** Peruvian maize, phenolic compounds, carotenoids, antioxidant capacity, biodiversity, *Zea mays* L.

## Abstract

The high diversity of the Peruvian Andean maize (*Zea mays* L.) represents a biological and genetic heritage relevant for food security, but few studies are targeted toward its characterization and consequent valorization and preservation. The objective of this study was to evaluate the potential of the Peruvian Andean maize race *Cabanita* with respect to its bioactive profiles (free and bound phenolic and carotenoid composition), physical characteristics, and *in vitro* antioxidant properties. Maize landraces with variable kernel pigmentation were collected from two provinces (Caylloma and Castilla) within the Arequipa region (among ten Andean sites) and the phytochemical profile was evaluated by Ultra High-Performance Liquid Chromatography with diode array detector (UHPLC-DAD). All maize samples were important sources of phenolic compounds mainly soluble *p*-coumaric and ferulic acid derivatives whereas anthocyanins were only detected in maize with partially red pigmented kernels. Major phenolic compounds in the bound phenolic fractions were ferulic acid and its derivatives along with *p*-coumaric acid. Carotenoid compounds including xanthophylls such as lutein, lutein isomers, and zeaxanthin were only detected in orange and white-yellow pigmented maize and are reported for the first time in Peruvian landraces. The multivariate analysis using Principal Components Analysis (PCA) revealed low variability of all data which may indicate a level of similarity among maize samples based on evaluated variables. However, maize grown in Caylloma province showed more homogeneous physical characteristics and higher yield, whereas higher phenolic contents and antioxidant capacity were observed in maize from Castilla. Samples CAY (yellow-pigmented kernel, Castilla) and COM (orange-pigmented kernel, Caylloma) had the highest total phenolic (246.7 mg/100 g dried weight basis, DW) and carotenoid (1.95 μg/g DW) contents among all samples. The variable Andean environmental conditions along with differences in farming practices may play a role and should be confirmed with further studies. Current results provide the metabolomic basis for future research using integrated omics platforms targeted toward the complete characterization of the ethnic-relevant maize race *Cabanita*.

## Introduction

Peru is in the central western region of South America and has been considered a megadiverse country since 2002 according to the Cancun Statement recognized by the United Nations (1, 2). Around 84 of the 117 life zones in the planet are found in Peru (3). The Andes Mountains located along its territory creating a complex geography with diversity of climates and ecosystems contains unique biodiversity hotspots (3, 4). These natural features together with the targeted selection and adaptation of several plant species by ancient Peruvian inhabitants over thousands of years have given rise to the high crop biodiversity found presently in Peru (3).

Maize (*Zea mays* L. ssp. *mays*) is one of the most important staple foods in Peru and is currently cultivated from regions at sea level to different Andean highland areas (5). The maize genetic diversity is classified into races which are defined as “a group of related individuals with enough characteristics in common to permit their recognition as a group” (6). A race of maize is comprised by landraces that are highly heterogeneous and genetically diverse open-pollinated dynamic populations developed under traditional farming systems (7–9). The landraces belonging to a same race show similar morphological characteristics, geographical distribution, ecological adaptation, and cultural importance (10). Peru and Mexico have been considered the two primary centers of maize domestication and both countries have the largest number of maize races in the world (11, 12). The Andean region is likely the zone with the major diversity of maize races in the world in terms of varied phenotypic characteristics such as kernel and ear morphology and pigmentations (13, 14). Nevertheless, the scientific information about Andean maize landraces is very limited in comparison with the significant research focused on Mexican germplasm (5).

The Peruvian maize genetic diversity was initially classified by Grobman et al. (15) who described 49 races and collected around 3931 accessions that have been preserved *ex situ* by the *Programa de Investigación y Proyección Social en Maíz* (PIPS *Maíz*) located at UNALM (*Universidad Nacional Agraria La Molina*, Lima, Peru). The Peruvian Ministry of Environment has conducted a new collection of maize landraces since 2013 with the aim to update the maize race classification (14). This is an ongoing process which is also considering the relevance of the cultural background and uses associated with each maize race. As a result, 52 races have been identified including new races and removing others (14).

The maize race *Cabanita* has been used as staple food since pre-Hispanic times in the southern Andean region of Arequipa in Peru. Its name likely derives from “Cabanaconde” which is the district name located in the Caylloma province where it is currently grown at around 3000 m above sea level on average. This amylaceous maize has importance at nutritional and economical levels for local indigenous communities since it is used in several traditional food preparations and sometimes represents their main economic income. In fact, the production of amylaceous maize has been partially responsible for the evident overcoming of rural poverty in Peru. However, the introduction of improved hybrids by some farmers may lead to a possible genetic erosion that should be avoided for preserving its genetic heterogeneity, which is the foundation for overall food crop resilience. The race *Cabanita* was not considered in the first classification probably due to the few samples collected from this region at that time; however, the current classification includes this race (14). It has been reported that its geographical distribution encompasses not only Arequipa, but also Moquegua and Tacna regions which are also located in the southern area of Peru (14).

The Latin American maize diversity has been highlighted as an important biological heritage that may play a fundamental essential role on the worldwide food security, and resilience to climate change (12). Recently, the genetic diversity of maize landraces has been related to variable bioactive profiles mainly phenolic and carotenoid compounds with different health-relevant functional properties (16). In case of Peru, research has been focused mostly on purple maize (*Kculli* race) because of its high content of anthocyanins (16, 17). Different studies have reported the phenolic composition of Peruvian purple maize and highlighted its associated bioactivity such as the antioxidant, antimicrobial, anti-hypertensive, and anti-cancer potential (18–23). However, limited information exists about the bioactive composition of other Peruvian maize races. Ranilla et al. (24) evaluated part of the wide Peruvian maize diversity in relation to its phenolic-antioxidant bioactive compounds and *in vitro* potential for the management of hyperglycemia and obesity. Traditional races from Arequipa including some samples (only 6) from the race *Cabanita* were evaluated in this study. The *Cabanita* group showed the second highest total ORAC (oxygen radical absorbance antioxidant capacity) value and α-amylase inhibitory activity relevant for hyperglycemia prevention after the *Kculli* group (purple-pigmented maize) (24). Hence, strategies integrating critical stakeholders for sustaining conservation coupled with advancing multidisciplinary research of Peruvian maize diversity should be developed.

Based on the previous research advances, the objective of the current study was to evaluate the diversity of the Peruvian Andean maize race *Cabanita* with respect to its bioactive profiles (free and bound phenolic and carotenoid composition), physical characteristics, and *in vitro* antioxidant properties. A collection of new *Cabanita* germplasm from ten Andean locations in Arequipa was performed. In addition, the information about the pre-harvest agronomical practices linked to the cultivation of *Cabanita* race was also documented. Results from this study could be further integrated to molecular approaches aimed at the complete characterization of the race *Cabanita* and the future development of targeted health and climate resilience targeted breeding applications for the benefit of indigenous Andean communities.

## Materials and methods

### Materials

**FIGURE 1:**
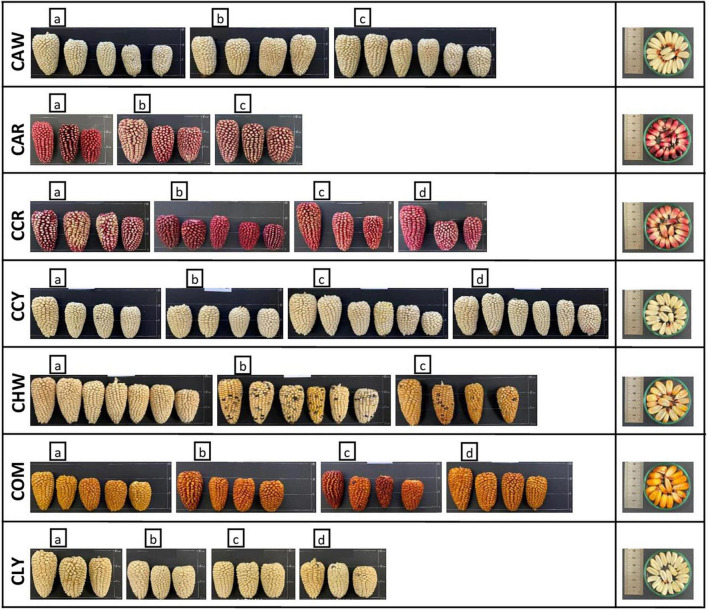
Ears and kernels of Cabanita maize samples collected from the Caylloma province located in the Arequipa region in Peru. Letters within each code location show the biological replicates.

**FIGURE 2:**
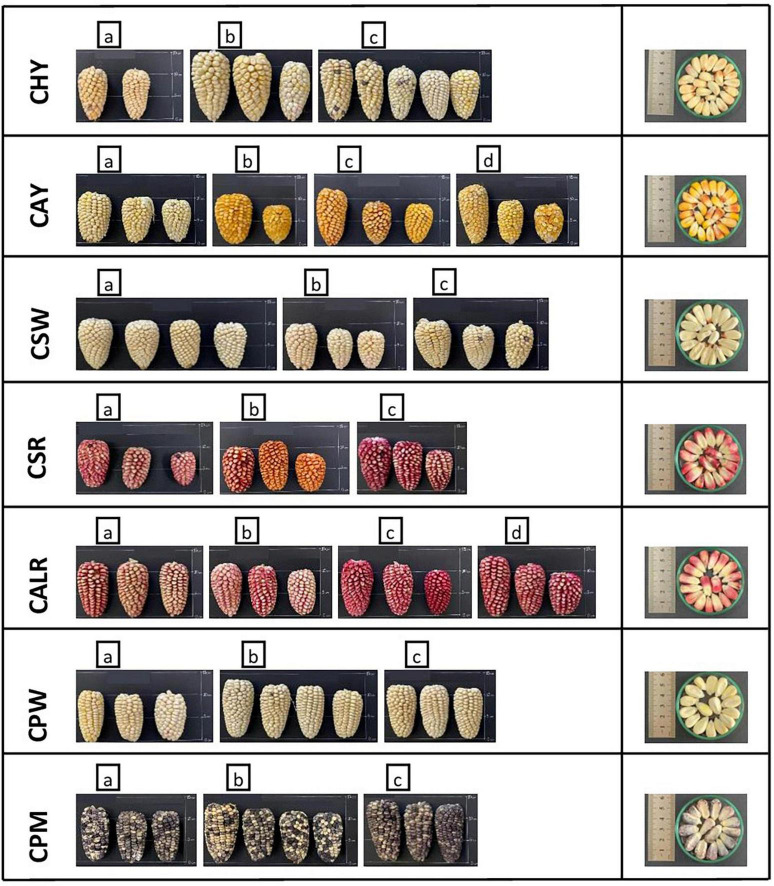
Ears and kernels of Cabanita maize samples collected from the Castilla province located in the Arequipa region in Peru. Letters within each code location show the biological replicates.

New germplasm of the maize race *Cabanita* was collected from two Andean provinces (Caylloma and Castilla) within the Arequipa region in Peru according to Ranilla et al. (24). The geographical information (coordinates, altitude, localities of origin) and the codification of maize samples are shown in Table 1. Around 9–20 and 9–12 ear units per maize type (red, white, yellow, or mixed pigmentation) and at commercial maturity were collected from Caylloma and Castilla provinces, respectively. At least five locations from each province were randomly sampled (total 10 locations) during the traditional harvest periods (May 2019 and June 2019 in case of maize from Caylloma and Castilla, respectively). All samples from Caylloma were collected from the Cabanaconde district (*Comision de Usuarios La Campiña*) whereas maize from Castilla was collected from three districts (Andahua, Ayo, Chachas) (Table 1). Maize ears (with husks) were mostly harvested directly from the plant. However, in some cases, ears were sampled when the maize plants were already cut and piled on the land or recently stored in the farmer’s warehouses. Samples were stored in cloth bags, protected from the light, and transported at 18–20°C to the laboratory in a maximum period of 34 h. Since ear samples were partially humid, the husks were eliminated and the ears were dried at environmental conditions with light protection until constant weight trying to mimic farmers’ postharvest practices but under controlled parameters (18.5–21.5°C, 29–34% of relative humidity). After drying, samples from each location and type were divided in 3–4 groups according to the similarity of their kernel pigmentation and ear characteristics. Each group corresponded to a biological replicate. Figures 1, 2 show pictures of ears and kernel samples from Caylloma and Castilla provinces, respectively. In addition, information about the agronomic pre-harvest practices used by local farmers for the cultivation of sampled maize plants was compiled (Supplementary Tables 1, 2 for samples from Caylloma and Castilla, respectively).

**TABLE 1:** Geographical information of Cabanita maize samples collected from Caylloma and Castilla provinces.

Province	District	Location	Code	Geographical coordinates	Altitude (masl*)
Caylloma	Cabanaconde	Auqui	CAW	S 15° 37′ 05.6′′	3110
			CAR	W 72° 00′ 37.8′′	
		Cusqui	CCR	S 15° 37′ 30.9′′	2964
			CCY	W 72° 00′ 04.1′′	
		Huancce-Tranca	CHW	S 15° 36′ 56.4′′	3332
				W 71°58′ 19.5′	
		Occollina-Tuntuiguita	COM	S 15° 37′ 41.6′′	3310
				W 71° 58′ 49.5′′	
		Liguay	CLY	S 15° 38′ 01.6′′	3266
				W 71° 58′ 49.0′′	
Castilla	Andahua	Huancarani	CHY	S 15° 29′ 50.7′′	3347
				W 72° 20′ 48.6′′	
		Ajocha	CAY	S 15° 29′ 57.2′′	3399
				W 72° 20′ 45.8′′	
	Ayo	Subna	CSW	S 15° 33′ 27.5′′	2845
			CSR	W 72° 14′ 16.3′′	
	Chachas	Alleachaya	CALR	S 15° 30′ 4.1′′	3070
				W 72° 16′ 10.7′′	
		Pulluguaya	CPW	S 15° 30′ 5.1′′	3043
			CPM	W 72° 16′ 14.4′′	

*Meters above sea level.

Dried ear maize samples and their corresponding kernels were used to evaluate the physical characteristics. Then, kernels were separated, pooled (per replicate) and stored at 5°C. A subsample of 50 g kernel was milled in a A11 Basic analytical mill (IKA, Germany) to a powdered flour (500 μm) and stored at –20°C until analysis.

### Reagents

Phenolic standards (ferulic acid, *p*-coumaric acid, cyanidin chloride, gallic acid), carotenoid standards (lutein, zeaxanthin), and the Folin-Ciocalteu reagent were from Sigma-Aldrich (St. Louis, MO, USA). The (±)-6-hydroxy-2,5,7,8-tetramethyl-chromane-2-carboxilic acid (Trolox), and the 2,2-diphenyl-1-picrylhydrazyl (DPPH^⋅^), and 2-2′-azino-bis(3ethylbenothiazoline-6-sulfonic acid) (ABTS^⋅+^) radicals were purchased from Sigma-Aldrich, USA.

### Moisture analysis and physical measurements

The moisture of kernels was determined by a gravimetric method at 105°C until constant weight (25). The physical characteristics were evaluated in both dried ears and kernels per replicate according to the descriptors defined by the *Programa de Investigación y Proyección Social en Maíz* (PIPS Maíz) (26) and CIMMYT (27). The weight (g), length (cm), tip, center and base diameters (cm), and the pith, rachis and cob diameters (cm) were measured in maize ear samples. Additionally, the number of rows per ear and the number of kernels per row were analyzed. In case of kernels, a row per ear with complete and sound grains was selected and 10 kernels were extracted from the central part. The kernel weight (g), length, width, and thickness (mm) were determined.

### Extraction of bioactive compounds from maize samples

#### Free and bound phenolic fractions

The free and bound phenolic fractions were extracted from the powdered maize samples following the methodology of Ranilla et al. (18) and Ranilla et al. (24). A mix of 0.1% HCl methanol/acetone/water (45:45:10, v/v/v) was used for the extraction of the free phenolic compounds. An alkaline hydrolysis with 3 N NaOH was applied on the free phenolic extraction residue for the release of the bound phenolic fraction (18, 24). Final aqueous extracts were kept at –20°C until further analysis.

#### Carotenoids

Preliminary trials based on the carotenoid extraction conditions reported by Egesel et al. (28) and Fuentealba et al. (29) were performed. The extraction of carotenoid compounds from powdered maize samples was applied according to Egesel et al. (28) with some modifications (30). An amount of 2–2.5 g of sample was mixed with 6 mL ethanol containing 0.01% (w/v) butylated hydroxytoluene (BHT), and then placed in a water bath at 85°C for 5 min. After this, 120 μL 80% KOH (w/v) was added, vortexed for 15 s, and further heated in the water bath for 10 min (with a 10 s mixing at minute 5). Samples were cooled in an ice bath and 3 mL of methanol:ethyl acetate (6:4, v/v) were added. The mixture was vortexed for 20 s and centrifuged at 2800 rpm for 10 min and the extract was stored under refrigeration (5°C). The resulting pellet was reextracted several times with same solvent volume until obtaining a clear final extract. Processed and stored extracts were pooled, first vacuum-evaporated, then concentrated under nitrogen atmosphere, and set to a known volume (3.5–5 mL) with methanol:ethyl acetate (6:4, v/v). All the procedure was carried out under light and oxygen protection as possible. Carotenoid extracts were analyzed by UHPLC immediately after the extraction process the same day.

### Analysis of the total phenolic contents

The Folin-Ciocalteu method was applied to measure the total phenolic contents (TPC) in the free and bound phenolic fractions from maize samples (31). The absorbance was recorded at 755 nm, and results were expressed as mg of gallic acid equivalents (GAE) per 100 g sample (dry weight basis, DW).

### Analysis of phenolic compounds by ultra high-performance liquid chromatography

A volume of 5 μL of the free and bound phenolic extracts previously filtered with a polyvinylidene difluoride filter (PVDF, 0.22 μm) was injected in an Ultimate 3000 RS UHPLC system (Thermo Fisher Scientific, Waltham, MA, USA) with a quaternary pump, autosampler, and coupled to a Vanquish diode array detector. Phenolic compounds were separated using a Kinetex C18 analytical column (100 × 2.1 mm i.d., 1.7 μm) with a Kinetex C18 guard column (5 × 2.1 mm i.d., 1.7 μm) (Phenomenex Inc., Torrance, CA, USA). The binary gradient elution with 0.1% formic acid and acetonitrile mobile phases, and chromatographic conditions were the same as used previously by Ranilla et al. (18) and Vargas-Yana et al. (32). The chromatograms and peaks were processed using the Chromeleon SR4 software version 7.2 (Thermo Fisher Scientific). Phenolic compounds were identified based on their retention times and spectral characteristics in comparison with external standards and the library spectra. The quantification was performed using calibration curves obtained with aglycone phenolic standards (*r*^2^ ≥ 0.9990). Peaks corresponding to phenolic acids (*p*-coumaric and ferulic acid) and their derivative compounds (with similar spectral features to those of pure standards, but with different retention times) were detected at 320 nm. The phenolic acid derivatives (*p*-coumaric and ferulic acid derivatives) were quantified using their corresponding phenolic acid calibration curves (*p*-coumaric and ferulic acid, respectively). Anthocyanins were detected at 525 nm and quantified as cyanidin chloride. Results were expressed as mg per 100 g sample DW.

### Analysis of carotenoid compounds by ultra high-performance liquid chromatography

Carotenoid extracts were filtered with a PVDF filter (0.22 μm) and injected (50 μL) in the same UHPLC system used for the analysis of phenolic compounds. Carotenoids were separated using a YMC carotenoid C30 analytical column (150 × 4.6 mm i.d., 3 μm) protected with a YMC C30 guard column (10 × 4.0 mm, 3 μm) (YMC CO., LTD, Kyoto, Japan). The chromatographic process was carried out at a flow rate of 1.7 mL/min, sample temperature of 10°C, column temperature of 30°C, and eluates were monitored at 450 nm. The reverse phase elution was performed using a ternary gradient elution with methanol (A), dichloromethane (B), and acetonitrile (C). The initial conditions were A/B/C (76/5/19) from 0 to 3.2 min, then solvent B was increased to 34% at 23.3 min (A/B/C: 52.8/34/13.2), and changed to initial conditions until the 25.1 min. The column was equilibrated for 2.9 more min (total run time of 28 min). The carotenoid peak identification was conducted by comparison of their retention times and spectral characteristics with those of external standards. Lutein and zeaxanthin were quantified using calibration curves (*r*^2^ ≥ 0.9900) built with pure carotenoid standards. Lutein isomers were quantified as lutein. Results were presented as μg per g sample DW.

### 2,2-Diphenyl-1-picrylhydrazyl radical inhibition antioxidant capacity

The 2,2-diphenyl-1-picrylhydrazyl radical (DPPH) antioxidant activity was determined in the free and bound phenolic extracts. In addition, the hydrophilic and lipophilic fractions from maize samples were obtained and analyzed. The extraction procedure reported by Fuentealba et al. (29) and Campos et al. (33) was followed with some modifications. For the hydrophilic fraction, a volume of 5 mL 80% methanol was added to 0.2 g of maize sample and mixed in an orbital shaker for 30 min at 230 rpm (environmental conditions). The homogenate was centrifuged at 5000 rpm for 15 min at 4°C and the supernatant (hydrophilic fraction) was separated and kept at – 20°C until use. The residual pellet was used for the extraction of the lipophilic fraction by the addition of 2 mL of dichloromethane, followed by shaking at 230 rpm for 30 min at environmental temperature. The mixture was centrifuged at 5,000 rpm, 4°C for 15 min. The supernatant (lipophilic fraction) was separated and stored at – 20°C until further analysis.

The DPPH method of Duarte-Almeida et al. (34) with modifications was applied. Sample extracts (40 μL) were mixed with 250 μL of diluted 317 μM DPPH solution in methanol. After 25 min of reaction at 25°C, the absorbance decrease was measured at 517 nm in a Biotek Synergy HTX microplate reader (Agilent, Santa Clara, CA, USA). A control with methanol or dichloromethane was included (for the aqueous hydrophilic, or lipophilic extracts, respectively). The antioxidant capacity was expressed as μmol Trolox equivalents per 100 g DW using a standard curve of Trolox (20–160 in methanol and 10–160 μM in dichloromethane, for the aqueous and hydrophilic, and lipophilic extracts, respectively).

### 2,2′-Azino-bis(3-ethylbenzothiazoline-6-sulfonic acid) radical cation (ABTS^⋅+^) inhibition antioxidant capacity

The ABTS antioxidant assay was determined in maize phenolic extracts (free and bound fractions), and the hydrophilic and lipophilic extracts according to Fuentealba et al. (29). A volume of 250 μL of 14.3 mM ABTS^⋅+^ diluted in 96% ethanol was added to 40 μL of sample extract. The reaction was carried out at 25°C for 30 min. Following which, the absorbance was recorded at 734 nm in a Biotek Synergy HTX microplate reader (Agilent). A control with methanol or dichloromethane was included (for the aqueous hydrophilic, or lipophilic extracts, respectively). Results were expressed as μmol Trolox equivalents per 100 g DW using same calibration curves as previously stated for the DPPH assay.

### Statistical analysis

All results (from 3 to 4 independent biological replicates) were expressed as means ± standard deviation. The data normality and variance homogeneity were evaluated with the Shapiro–Wilk and Levene tests, respectively (α = 0.05). Data were subjected to an analysis of variance (ANOVA) with the HSD Tukey test for multiple comparisons or the Kruskall–Wallis test and the Dunn’s test with Bonferoni arrangement for mean comparisons. The R software version 4.0.4 (R Foundation for Statistical Computing, Vienna, Austria) was used. The unsupervised multivariate principal component analysis (PCA) was also applied to all data using the Unscrambler^®^ X software version 10.4 (Aspen Technology, Inc., Bedford, MA, USA). Further, Pearson correlations among all data were explored using the Statgraphics Centurion XVI software (StatPoint Inc., Rockville, MD, USA).

## Results and discussion

### Environmental conditions and pre-harvest agricultural management associated with the cultivation of evaluated maize samples

The environmental conditions (maximum and minimum temperature, rainfall, and relative humidity) linked to the period of maize cultivation (August-2018 to May-2019) from each district of sample’s origin are shown in Supplementary Figures 1–4 (Cabanaconde, Andahua, Chachas, and Ayo districts, respectively). The maximum temperature was overall less variable than minimum temperature ranges in all districts. The Andahua district (Castilla province) showed the lowest minimum (2.8–6.0°C) and maximum (16–18.7°C) temperature ranges, whereas the Ayo district (Castilla province) had the highest minimum (6–13°C) and maximum (27.6–29.9°C) temperature values. The relative humidity variability was similar in the Andahua and Chachas districts (Castilla province), but their average values were lower (57.7 and 54.7%, respectively) than values shown in the Cabanaconde and Ayo districts (81.7 and 70.1%, respectively). The precipitation levels were very low in all districts, showing only some millimeters per day in January and February in all cases. The ultraviolet (UV) index was measured during the collection of maize samples in most of the localities from both provinces (data not shown). Values varied from 7.5 to 10 (at noon time approximately) and are classified as “high to very high” according to the World Health Organization scale (35). Based on these climate characteristics, Cabanita maize plants might have been exposed to variable abiotic stress factors. Garcia et al. (36) have also reported similar environmental harsh conditions in Andean highlands.

Supplementary Tables 1, 2 show information about the agricultural management practices applied by farmers during the cultivation of *Cabanita* maize evaluated in the current research. Maize had a vegetative stage of 8 months and is sown once a year around the same period in both provinces. Farmers from both provinces follow similar farming practices such as the exchange of selected maize kernels among their neighboring communities to be used as seeds, the use of organic fertilizers for soil preparation, and a fallow stage of 3–4 months. The traditional seed exchange systems have shown to generate a flow of genes that is crucial for the *in situ* agrobiodiversity conservation (37). Likewise, fallow systems have been used in traditional agriculture to regenerate and improve soil fertility through the enhancement of soil organic matter following continuous cropping specially in the smallholder farming systems (38, 39). This indicates that farmers from this southern Peruvian Andean region generally preserve ancestral agricultural techniques relevant for biodiversity conservation and overall cultivate maize under organic conditions (control of weeds and pests manually or with flood irrigation, respectively).

However, some differences have been observed in relation to the presence of previous or simultaneous crops, such as the use of organic fertilizers during maize growth, and the origin of the water for irrigation. Caylloma lands seem to be exclusively used for *Cabanita* maize cultivation, the soil fertilization during the maize growth is not frequent, and some *Medicago* species may emerge at this stage (Supplementary Tables 1, 2). In contrast, farmers from Castilla reported previous crops in same lands used for maize cultivation including tuberous species, legumes, and cereals (oat, quinoa, barley), the soil is fertilized with manure during the maize growth, and other plant species such as certain legumes (*Vicia faba* and *Medicago sativa*) are simultaneously grown with maize plants. Legumes including *Vicia fava* have shown high potential for nitrogen fixation, and other legume species have been already used in combination with grasses to improve the soil nutrient regeneration and its physical characteristics in the Peruvian highlands during the fallow stages (40, 41). In addition, some farmers from Caylloma stated out the use of cattle manure or domestic poultry manure, whereas other farmers from Castilla used guinea pig residues besides the cattle manure for soil preparation (information not completely provided). The type of crop rotation and organic fertilization may determine differences in the rhizosphere ecosystems influencing the soil microbiome diversity and composition, and consequently impacting its agronomic performance (42, 43). Additionally, the levels of irrigation have shown to affect the yield and nutritional quality of maize (44). No information about the soil composition and the irrigation frequency from each location were obtained in the current study. The effect of the specific agricultural management differences applied by farmers and the likely interaction with the Andean environmental conditions on soil characteristics along with the influence of these factors on maize quality and composition should be better investigated in future studies.

### Physical characteristics of maize samples

Quantitative physical characteristics were measured in dried ears and kernels of *Cabanita* maize as shown in Table 2. In general, maize from the Castilla province showed higher variability in their physical descriptors than samples from Caylloma according to the statistical analysis. Maize ears from Caylloma were characterized for showing higher length, center diameter values, along with higher pith, rachis, and cob diameters than ears from Castilla. Samples CHW and CAW had the highest length and center diameters among all samples (12.4 and 6.0 cm, respectively). No statistical differences were observed in the tip and base diameters in maize from both provinces. However, the base diameter ranges were higher than tip values indicating that *Cabanita* maize ears exhibit a conic-cylindrical shape.

**TABLE 2:** Physical characteristics of Cabanita maize samples (ear and kernel) from Caylloma and Castilla provinces.

Characteristic	Caylloma							Castilla						
	CCR	CCY	COM	CAW	CAR	CLY	CHW	CHY	CAY	CSW	CSR	CPW	CPM	CALR
**Kernel**														
Weight (g)	0.4 ± 0.1bc	0.4 ± 0.0bc	0.5 ± 0.0ab	0.4 ± 0.0bc	0.4 ± 0.0bc	0.4 ± 0.0bc	0.5 ± 0.0ab	0.4 ± 0.1bc	0.3 ± 0.1c	0.5 ± 0.1ab	0.6 ± 0.1ab	0.6 ± 0.0ab	0.6 ± 0.1a	0.6 ± 0.1ab
Length (mm)	16.2 ± 1.1a	15.4 ± 1.1a	17.5 ± 0.5a	17.0 ± 0.6a	16.0 ± 1.0a	16.5 ± 1.0a	16.8 ± 1.2a	15.3 ± 0.6a	14.8 ± 2.4a	17.6 ± 2.2a	16.6 ± 2.5a	16.9 ± 0.7a	16.9 ± 0.1a	15.9 ± 0.6a
Width (mm)	7.7 ± 0.8c	8.4 ± 0.3abc	8.5 ± 0.3abc	8.0 ± 0.7bc	8.3 ± 0.4abc	8.1 ± 0.6bc	8.1 ± 0.2bc	9.1 ± 0.6abc	7.4 ± 0.3c	9.1 ± 0.6abc	8.9 ± 1.3abc	9.6 ± 0.4ab	10.0 ± 0.8a	9.4 ± 0.6ab
Thickness (mm)	5.5 ± 0.1a	5.4 ± 0.3a	5.4 ± 0.3a	5.0 ± 0.3a	5.2 ± 0.2a	5.3 ± 0.3a	5.5 ± 0.1a	6.0 ± 0.5a	5.3 ± 0.3a	5.5 ± 0.2a	5.6 ± 1.2a	6.0 ± 0.4a	5.7 ± 0.2a	6.1 ± 0.2a
**Ear**														
Weight (g)	159.6 ± 29.3a	136.1 ± 13.4ab	155.2 ± 10.1a	162.7 ± 28.9a	154.4 ± 19.3ab	134.5 ± 21.3ab	177.8 ± 15.7a	97.9 ± 9.0bc	69.4 ± 14.3c	122.1 ± 29.4abc	125.6 ± 23.6ab	139.3 ± 28.7ab	170.1 ± 16.2a	134.9 ± 11.6ab
Length (cm)	10.9 ± 1.4ab	9.9 ± 1.2bc	10.6 ± 0.6ab	11.0 ± 0.3ab	10.9 ± 0.1ab	10.6 ± 1.2ab	12.4 ± 0.9a	10.3 ± 0.2abc	8.1 ± 0.1c	9.2 ± 1.0bc	9.3 ± 0.9bc	10.7 ± 0.9ab	11.4 ± 0.8ab	10.5 ± 0.4ab
Tip diameter (cm)	2.3 ± 0.6a	2.6 ± 0.4a	2.4 ± 0.3a	2.9 ± 0.6a	2.8 ± 0.3a	2.8 ± 0.1a	2.2 ± 0.1a	2.2 ± 0.1a	3.0 ± 0.5a	2.6 ± 0.2a	3.0 ± 0.0a	2.8 ± 0.4a	3.2 ± 0.8a	2.7 ± 0.1a
Center diameter (cm)	6.0 ± 0.3a	5.9 ± 0.2a	5.9 ± 0.3a	6.0 ± 0.4a	5.8 ± 0.3ab	5.8 ± 0.3a	5.9 ± 0.3a	4.9 ± 0.2c	5.1 ± 0.3bc	5.6 ± 0.1abc	5.5 ± 0.3abc	5.5 ± 0.3abc	5.8 ± 0.1ab	5.5 ± 0.1abc
Base diameter (cm)	4.4 ± 0.2a	3.9 ± 0.8a	4.5 ± 0.3a	4.2 ± 0.3a	4.3 ± 0.5a	4.4 ± 0.2a	4.5 ± 0.2a	4.1 ± 0.5a	4.1 ± 0.2a	4.6 ± 0.4a	4.2 ± 0.2a	4.4 ± 0.4a	4.2 ± 0.7a	4.3 ± 0.1a
Pith diameter (cm)	1.1 ± 0.2abc	1.0 ± 0.3abc	1.1 ± 0.2abc	1.4 ± 0.2a	1.2 ± 0.3abc	1.0 ± 0.1abc	1.2 ± 0.1ab	0.9 ± 0.1abc	1.0 ± 0.2abc	0.7 ± 0.1c	0.7 ± 0.1c	0.8 ± 0.2bc	0.9 ± 0.0bc	0.8 ± 0.0bc
Rachis diameter (cm)	2.0 ± 0.3abc	1.9 ± 0.3abc	1.9 ± 0.1abc	2.4 ± 0.4a	2.2 ± 0.3ab	2.0 ± 0.1abc	2.2 ± 0.2ab	1.8 ± 0.1abc	2.0 ± 0.2abc	1.6 ± 0.4bc	1.7 ± 0.1bc	1.6 ± 0.2c	1.9 ± 0.1abc	1.8 ± 0.2bc
Cob diameter (cm)	2.9 ± 0.3abcd	2.8 ± 0.4abcd	2.7 ± 0.1abcd	3.3 ± 0.5a	3.2 ± 0.4ab	2.7 ± 0.2abcd	3.1 ± 0.4abc	2.6 ± 0.1abcd	2.9 ± 0.2abcd	2.2 ± 0.3d	2.4 ± 0.1bcd	2.3 ± 0.2cd	2.6 ± 0.2abcd	2.5 ± 0.2bcd
Number of rows/ear	17.6 ± 1.4abc	18.0 ± 0.6ab	17.0 ± 0.5abcde	18.9 ± 2.2a	17.0 ± 0.9abcde	17.2 ± 0.4abcd	18.6 ± 1.8a	14.0 ± 1.0de	18.2 ± 1.6a	14.5 ± 0.8cde	14.1 ± 1.2de	14.7 ± 1.6bcde	14.6 ± 1.5bcde	13.8 ± 1.0e
Number of kernels/row	19.3 ± 2.5abc	18.9 ± 2.0abc	18.8 ± 1.9abc	20.5 ± 0.5ab	19.6 ± 0.5abc	18.3 ± 1.6abcd	21.2 ± 0.6a	17.1 ± 1.0abcd	15.0 ± 1.0d	17.2 ± 0.8abcd	15.3 ± 1.9cd	17.6 ± 1.4abcd	20.0 ± 1.5ab	16.6 ± 0.7bcd

Different letters within the same row indicate significant statistical differences (p < 0.05).

The yield-relevant physical parameters such as the ear weight, number of rows per ear and number of kernels per row were higher in maize from Caylloma (136.1–177.8 g, 17.0–18.9, 18.3–21.2; respectively) than ranges observed in samples from Castilla (69.4–170.1 g, 13.8–18.2, 15–20; respectively). Maize CHW, followed by CAW, CCR, and COM all from Caylloma exhibited the highest ear weights (177.8, 162.7, 159.6, and 155.2 g, respectively). In case of Castilla, only CPM sample had a comparable ear weight (170.0 g) as the observed ranges in maize from Caylloma. A strong positive correlation was obtained between the number of rows per ear and the ear weight (*r* = 0.7767, *p* < 0.05). Soil characteristics linked to different fallow and fertilization systems have shown to impact yield in several crops including maize (45, 46). As stated previously, some differences have been observed during the pre-harvest agricultural management of *Cabanita* cultivation that may determine better yields in maize grown in Caylloma than in Castilla. Other factors such as genetic, and environmental conditions might be also involved and need further research.

In relation to the physical characteristics of kernels, no differences were found in the length and thickness of samples from both provinces. Nevertheless, the weight and width values were more variable in samples from Castilla than in maize kernels from Caylloma. The kernel width and weight ranged from 7.7 to 8.5 mm and from 0.4 to 0.5 g, respectively in Caylloma maize. In case of Castilla, the width and weight results varied from 7.4 to 10.0 mm and from 0.3 to 0.6 g, respectively. Sample CPM had the highest width and weight values among all samples.

The variability of kernel pigmentations as shown in Figures 1, 2 may be related to differences in the bioactive compound contents and profiles as will be further discussed in next sections. In contrast to a previous investigation where only some *Cabanita* maize samples with white-yellow to mixed yellow-red-pigmented kernels were evaluated (24), a more complete *in situ* collection of *Cabanita* maize samples with variable kernel pigmentations has been performed in the current study. The wide phenotypic diversity of the Peruvian maize races has been reported by the Peruvian Ministry of Environment (14) with limited information about the race *Cabanita*. Results from current study contributes to the physical characterization of this ethnically important maize race for southern Peruvian Andean communities.

### Phenolic bioactive composition

The phenolic profiles and contents from *Cabanita* maize samples are shown in Table 3. The free phenolic fractions were mostly rich in *p*-coumaric acid derivatives (3.5–8.2 mg/100 g DW), followed by ferulic acid derivatives (1.3–5.9 mg/100 g DW), and free forms of *p*-coumaric and ferulic acids (0.2–0.9 and 0.5–0.8 mg/100 g DW, respectively). Flavonoids such as anthocyanins were only detected in maize with partially red or purple-pigmented kernels (CCR, CAR, CSR, CPM, CALR) at higher concentrations (0.9–5.3 mg/100 g DW) than in white maize with some variegated-purple kernels (0.1 and 0.2 mg/100 g DW for samples CHY and CHW, respectively). The bound phenolic fraction represented on average 95% of the TPC (free + bound) similarly as in previous studies with Peruvian and Chilean maize landraces (24, 47). The major bound phenolic compounds were ferulic acid (98.6–195.1 mg/100 g DW) and ferulic acid derivatives (15.0–28.2 mg/100 g DW). Bound *p*-coumaric acid was found at lower concentrations (10.5–19.3 mg/100 g DW). Ranilla et al. (24) first evaluated the phenolic composition in 6 *Cabanita* maize accessions from the same Peruvian region reporting free *p*-coumaric acid and ferulic acid derivatives ranges below current results (2.6–5.5 and 0.2–0.9 mg/100 g DW, respectively).

**TABLE 3:** Ultra high-performance liquid chromatography (UHPLC) phenolic profiles and contents (mg/100 g DW) of Cabanita maize samples from Caylloma and Castilla provinces.

Compound	Caylloma							Castilla						
	CCR	CCY	COM	CAW	CAR	CLY	CHW	CHY	CAY	CSW	CSR	CPW	CPM	CALR
**Anthocyanins**														
Free (Total)	2.8 ± 1.6ab	ND	ND	ND	2.9 ± 1.7ab	ND	0.2 ± 0.2b	0.1 ± 0.1b	ND	ND	0.9 ± 0.9ab	ND	5.3 ± 0.4a	0.9 ± 0.5ab
***P*-coumaric acid**														
Free	0.3 ± 0.0ab	0.3 ± 0.1ab	0.5 ± 0.1ab	0.3 ± 0.0ab	0.2 ± 0.0b	0.4 ± 0.1ab	0.5 ± 0.1ab	0.3 ± 0.1ab	0.9 ± 0.4a	0.8 ± 0.1a	0.6 ± 0.1ab	0.3 ± 0.0ab	0.3 ± 0.0ab	0.3 ± 0.0ab
Bound	11.5 ± 3.0a	11.7 ± 2.4a	10.5 ± 4.4a	11.5 ± 1.2a	11.9 ± 5.2a	10.6 ± 4.0a	13.4 ± 3.6a	14.0 ± 3.6a	19.3 ± 4.5a	10.7 ± 1.6a	15.8 ± 3.6a	11.3 ± 3.3a	11.0 ± 1.7a	13.8 ± 1.8a
Total	11.8 ± 3.0ab	12.0 ± 2.4ab	10.9 ± 4.5b	11.8 ± 1.2ab	12.1 ± 5.2ab	10.9 ± 4.0b	13.9 ± 3.6ab	14.3 ± 3.6ab	20.2 ± 4.8a	11.5 ± 1.5ab	16.3 ± 3.6ab	11.5 ± 3.3ab	11.3 ± 1.7ab	14.2 ± 1.8ab
***P*-coumaric acid derivatives**														
Free*	4.4 ± 0.7ab	4.9 ± 1.4ab	6.3 ± 0.7ab	3.5 ± 0.2b	3.7 ± 0.1ab	4.8 ± 0.4ab	6.0 ± 0.6ab	4.4 ± 0.3ab	8.2 ± 2.4a	7.5 ± 1.0ab	7.4 ± 0.6ab	4.0 ± 0.5ab	4.2 ± 1.0ab	3.7 ± 0.1ab
**Ferulic acid**														
Free	0.7 ± 0.2a	0.6 ± 0.1a	0.6 ± 0.1a	0.6 ± 0.2a	0.6 ± 0.2a	0.5 ± 0.1a	0.6 ± 0.1a	0.6 ± 0.1a	0.7 ± 0.1a	0.8 ± 0.1a	0.8 ± 0.1a	0.6 ± 0.0a	0.6 ± 0.0a	0.7 ± 0.1a
Bound	152.6 ± 27.4ab	161.8 ± 46.2ab	98.0 ± 36.6b	170.7 ± 25.8ab	139.44 ± 42.4ab	151.4 ± 38.9ab	161.1 ± 28.7ab	177.0 ± 42.5ab	189.0 ± 38.1a	192.0 ± 9.9a	188.6 ± 39.5ab	195.1 ± 25.2a	163.9 ± 5.9ab	192.4 ± 31.5a
Total	153.3 ± 27.4ab	162.3 ± 46.2ab	98.6 ± 36.5b	171.3 ± 25.8ab	140.1 ± 42.4ab	151.9 ± 38.9ab	161.7 ± 28.7ab	177.7 ± 42.6ab	189.7 ± 38.2a	192.8 ± 9.9a	188.4 ± 39.4ab	195.7 ± 25.2a	164.5 ± 5.9ab	193.1 ± 31.6a
**Ferulic acid derivatives**														
Free	2.0 ± 0.5ab	1.7 ± 0.5ab	3.4 ± 1.0ab	1.3 ± 0.4b	1.4 ± 0.5b	1.6 ± 0.1ab	3.0 ± 0.5ab	3.1 ± 0.4ab	5.9 ± 1.7a	3.3 ± 0.7ab	2.8 ± 0.8ab	2.5 ± 0.7ab	1.3 ± 0.2b	3.4 ± 0.6ab
Bound	16.5 ± 2.9a	17.6 ± 4.7a	15.0 ± 6.0a	18.1 ± 2.4a	16.4 ± 5.1a	16.3 ± 3.6a	19.7 ± 1.8a	24.0 ± 7.6a	22.7 ± 2.9a	22.0 ± 2.0a	18.7 ± 3.1a	28.2 ± 1.7a	26.7 ± 1.8a	16.4 ± 5.1a
Total	18.5 ± 2.7a	19.3 ± 4.3a	18.4 ± 6.0a	19.4 ± 2.1a	17.8 ± 4.9a	17.9 ± 3.7a	22.8 ± 2.2a	27.1 ± 7.2a	28.6 ± 2.5a	25.3 ± 2.5a	21.6 ± 3.1a	30.7 ± 2.3a	28.0 ± 2.0a	27.0 ± 3.9a
**Total phenolic acids** (Free)	7.4 ± 0.8ab	7.5 ± 1.8ab	10.8 ± 1.2ab	5.7 ± 0.7b	6.0 ± 0.5b	7.2 ± 0.6ab	10.2 ± 0.7ab	8.5 ± 0.9ab	15.6 ± 3.5a	12.4 ± 1.8ab	11.6 ± 1.2ab	7.4 ± 0.5ab	6.5 ± 1.0ab	9.4 ± 1.0ab

Different letters within the same row indicate significant statistical differences (p < 0.05). ND, non-detected.

Different soluble conjugated phenolic compounds have been found in cereals including maize (48, 49). Hydroxycinnamic acid amides (HCAAs) mainly derived from *p*-coumaric and ferulic acids such as *N,N*′-di-*p*-coumaroylspermine, *N*-*p*-coumaroyl-*N*′-feruloylputrescine and *N,N*′-diferuloylputrescine (DFP) have been detected in the soluble fraction of the yellow maize variety *Amagrano* from Germany (50). These phenolic amides were found at higher concentrations (∼14.8–18.03 mg/100 g DW) than the free forms of *p*-coumaric and ferulic acids (∼3.5–3.9 mg/100 g DW) whereas only traces of mono- and dihydroxycinnamoyl glycerides have been detected (50). Likewise, DFP, feruloylputrescine, cinnamoylputrescine and caffeoylputrescine (0.1–2.4 mg/100 g DW in total in whole grain) have been found in a wide diversity of Mexican maize landraces and have been proposed as taxonomic markers (51). The concentrations of hydroxycinnamic acid derivatives found in this investigation are consistent with ranges reported in above studies. Additional analyses with better analytical detection are needed to confirm the identification of soluble phenolic compounds in *Cabanita* maize. HCAAs are more concentrated in the outer layers (pericarp and aleurone) of maize kernel and have been shown to be part of the natural plant defense against pests and other biotic and abiotic stress factors (51–53). The anthocyanin concentrations found in Cabanita maize agree with the amounts reported by Paulsmayer et al. (54) (0.3–12.8 mg/100 g DW of total anthocyanins with a HPLC method) in diverse pink aleurone maize germplasm from Mexico and the United States with similar kernel pigmentations as those observed in *Cabanita* maize samples (Figures 1, 2). Partially red-pigmented kernels in some evaluated samples indicate lower anthocyanin contents in comparison to purple pericarp-pigmented maize (18).

In case of the bound phenolic fraction, overall higher concentrations of ferulic acid, and ferulic acid derivatives have been detected in current *Cabanita* maize than in a previous study with Peruvian germplasm (107–139 and 17–21 mg/100 DW) (24). In addition, comparable bound *p*-coumaric acid values (19.6–22.5 mg/100 g DW) has been obtained in the same research as those found in this current research. Discrepancy of current results from previously reported phenolic values in same maize race may be related to differences in the pre-harvest practices and sampling conditions and is explained at the end of this section. Ferulic acid and *p*-coumaric acid have also been highlighted as the major bound or insoluble phenolic compounds in other maize landraces and different mature cereal grains such as wheat, rice, and barley (47, 55–57). Other minor bound phenolic compounds in maize comprise of diferulic acids such as 8-*O*-4′-diferulic acid, 5,5′-diferulic acid, and 8,5′-diferulic acid along with triferulic acids (50). It is most likely that ferulic acid derivatives observed in *Cabanita* maize are diferulic or triferulic acids, but this should be further confirmed with mass spectrometry analyses. Total diferulic acid contents found by Zavala-López et al. (58) in several modern and traditional maize hybrids were lower (4.3–13.9 mg/100 g DW) than ferulic acid derivatives quantified in this research (15.0–28.2 mg/100 g DW).

Table 4 shows the TPC determined in the free and bound phenolic fractions using the UHPLC and the Folin-Ciocalteu methods. Bound phenolic contents were overall similar with both methods; however, higher free TPC values were detected with the spectrophotometric method than by UHPLC. This may be explained by the presence of other non-phenolic soluble reducing compounds in evaluated maize extracts. It has been reported that the Folin-Ciocalteu method lacks specificity because of several interfering compounds including proteins, amino acids, aromatic amines, sugars, organic acids, among other organic compounds (59). Overestimated free phenolic contents have been found in yellow corn flour which was related to the presence of soluble interfering compounds such as proteins and reducing sugars (60).

**TABLE 4:** Total phenolic contents of Cabanita maize samples from Caylloma and Castilla provinces.

Compound	Caylloma							Castilla						
	CCR	CCY	COM	CAW	CAR	CLY	CHW	CHY	CAY	CSW	CSR	CPW	CPM	CALR
**UHPLC TPC (mg/100 g DW)**														
Free	10.3 ± 2.4bcd	7.5 ± 1.8cd	10.8 ± 1.2bc	5.7 ± 0.7d	8.9 ± 1.9bcd	7.2 ± 0.6cd	10.4 ± 0.6bcd	8.7 ± 0.9bcd	15.6 ± 3.5a	12.4 ± 1.8ab	12.6 ± 0.6ab	7.4 ± 0.5cd	11.8 ± 0.8abc	10.3 ± 1.4bcd
Bound	180.6 ± 31.8ab	191.1 ± 52.1ab	123.4 ± 45.6b	200.3 ± 29.3ab	167.7 ± 51.3ab	178.3 ± 44.3ab	194.2 ± 32.7ab	215.0 ± 49.8ab	231.1 ± 44.7a	224.7 ± 10.3ab	223.0 ± 42.4ab	234.6 ± 28.1a	201.6 ± 8.2ab	229.8 ± 36.2a
Total	190.9 ± 31.2ab	198.6 ± 51.3ab	134.3 ± 45.9b	206.0 ± 29.1ab	176.6 ± 49.5ab	185.5 ± 44.7ab	204.6 ± 33.0ab	223.6 ± 49.6ab	246.7 ± 45.2a	237.1 ± 10.7ab	235.6 ± 41.8ab	242.0 ± 28.6ab	213.3 ± 7.9ab	240.1 ± 37.1ab
**Folin–Ciocalteu TPC (mg GAE/100 g DW)**														
Free	44.4 ± 10.3ab	40.7 ± 4.0ab	47.3 ± 1.2ab	37.2 ± 2.8b	42.8 ± 2.7ab	40.6 ± 1.0ab	42.8 ± 3.8ab	50.8 ± 6.6ab	63.4 ± 5.3a	49.4 ± 4.7ab	48.9 ± 0.7ab	39.4 ± 1.0ab	56.2 ± 4.7ab	47.3 ± 2.8ab
Bound	193.1 ± 31.4a	213.8 ± 27.4a	154.2 ± 51.9a	222.6 ± 14.2a	180.2 ± 59.7a	192.5 ± 53.4a	204.7 ± 27.0a	234.4 ± 33.3a	226.9 ± 50.0a	221.2 ± 7.0a	230.7 ± 48.7a	251.8 ± 32.9a	215.8 ± 11.2a	232.5 ± 26.9a
Total	237.5 ± 35.0a	254.5 ± 30.1a	201.5 ± 51.3a	259.8 ± 16.8a	222.9 ± 57.4a	233.1 ± 54.3a	247.5 ± 23.4a	285.3 ± 33.9a	290.4 ± 48.0a	270.7 ± 6.9a	279.6 ± 49.2a	291.1 ± 32.7a	272.0 ± 13.8a	279.8 ± 28.7a

Different letters within the same row indicate significant statistical differences (p < 0.05).

More statistical variability was observed in the UHPLC results than in those obtained by the spectrophotometric method (TPC). In general, phenolic levels in the free fraction were not much affected by the province of origin. UHPLC free phenolic ranges were 8.7–15.6 mg/100 g DW and 5.7–10.8 mg/100 g DW, for Castilla and Caylloma samples, respectively. However, *Cabanita* maize from Castilla had higher bound phenolic contents than Caylloma samples. The total bound phenolic values were 201.6–234.6 and 123.4–200.3 mg/100 g DW for Castilla and Caylloma samples, respectively. A similar trend was observed in the TPCs (free + bound) with both methods. Sample CAY (Castilla) showed the highest UHPLC TPCs followed by CPW sample (Castilla) (246.7 and 242.0 mg/100 g DW, respectively) whereas COM (Caylloma) maize had the lowest concentrations (134.3 mg/100 g DW). CAY maize also exhibited the highest total *p*-coumaric acid and *p*-coumaric acid derivatives along with high ferulic acid contents similarly as in other Castilla samples (CPW, CALR, CSW).

The TPC (free + bound) range of this investigation (201.5–291.1 mg GAE/100 g DW) was almost twofold higher than results obtained previously in kernels from the same Peruvian race (133.5–158.4 mg GAE/100 g DW) (24). This may be explained by differences in the sampling procedure. In current study, the ears collection was generally performed directly from the plant or recently harvested plants. Ranilla et al. (24) evaluated stored kernels from germplasm bank or farmers’ warehouses who generally stored dried kernels under indeterminate time and after a sun-drying process for several weeks on the land. The storage time have shown to decrease the phenolic concentrations in sorghum grain and flour (61). Other factors related to the cultivation and harvesting practices also influence the phenolic contents in cereal grains and might also explain observed differences (62). The TPC range obtained in this study was comparable to that reported by De la Parra et al. (63) (243.8–320.1 mg GAE/100 g DW, samples from the United States) and higher than results found in maize landraces with variable pigmentations from Chile (132.2–262.5 mg GAE/100 g DW) (47), Mexico (77.3–123.6 mg GAE/100 g DW) (64) and India (102.3–206.4 mg GAE/100 g DW) (65).

### Carotenoid composition

The total carotenoid contents in evaluated samples were lower (0.09–1.95 μg/g DW) than levels of phenolic compounds (Table 5). Xanthophylls such as lutein, lutein isomers and zeaxanthin were found in orange-pigmented (COM) and white-yellow samples (CCY, CLY, CHW, CAY, CPW). No carotenoids have been detected in most partially red-pigmented kernels (CCR, CAR, CALR) and some white maize (CHY, CSW). Lutein and lutein isomers were the major carotenoids in all *Cabanita* maize except in CHW sample where higher zeaxanthin (0.39 μg/g DW) than lutein levels (∼0.20 μg/g DW) were found. Sample COM (Caylloma) had the highest total carotenoid values (1.95 μg/g DW), followed by CAY (0.85 μg/g DW, Castilla) and CHW (0.57 μg/g DW, Caylloma). Samples CSR and CPM (with red-orange and purple variegated-pigmented kernels, respectively) which showed anthocyanin compounds (Table 3), also had carotenoids but at lower concentrations (0.09–0.31 μg/g DW).

**TABLE 5:** Carotenoid profiles and contents (μg/g DW) of Cabanita maize samples from Caylloma and Castilla provinces.

Compounds	Caylloma							Castilla						
	CCR	CCY	COM	CAW	CAR	CLY	CHW	CHY	CAY	CSW	CSR	CPW	CPM	CALR
Lutein	ND	ND	1.37 ± 0.52a	ND	ND	ND	0.10 ± 0.07ab	ND	0.24 ± 0.12ab	ND	0.15 ± 0.13ab	0.07 ± 0.12b	0.04 ± 0.02b	ND
Lutein isomers	ND	0.10 ± 0.08ab	0.23 ± 0.17ab	ND	ND	0.12 ± 0.05ab	0.08 ± 0.06ab	ND	0.36 ± 0.24a	ND	0.11 ± 0.06ab	0.11 ± 0.02ab	0.03 ± 0.03b	ND
Zeaxanthin	ND	ND	0.36 ± 0.09a	ND	ND	ND	0.39 ± 0.61a	ND	0.25 ± 0.23a	ND	0.05 ± 0.06a	ND	0.01 ± 0.01a	ND
Total carotenoids	ND	0.10 ± 0.08b	1.95 ± 0.70a	ND	ND	0.12 ± 0.05ab	0.57 ± 0.60ab	ND	0.85 ± 0.52ab	ND	0.31 ± 0.24ab	0.17 ± 0.13ab	0.09 ± 0.05b	ND

Different letters within the same row indicate significant statistical differences (p < 0.05). ND: Non-detected.

Lutein and zeaxanthin have also been shown as the major carotenoid compounds specially in white, yellow, and red-pigmented maize kernels. Kuhnen et al. (66) reported lower zeaxanthin and lutein ranges (0.08–0.18 μg/g DW and 0.03–0.07 μg/g DW, respectively) in white Brazilian maize landraces compared with yellow landraces (0.07–7.05 μg/g DW and 0.48–3.69 μg/g DW, for zeaxanthin and lutein, respectively). White maize landraces from Malawi have shown ranges of 0.14–0.18 and 0.05–0.11 μg/g DW for lutein and zeaxanthin, respectively (67). In contrast, higher contents of both xanthophylls (4.7–34.97 and 2.36–21.18 for lutein and zeaxanthin, respectively) have been obtained by Uarrota et al. (68) in yellow Brazilian landraces along with moderate ranges of different pro-vitamin A carotenoids. Further, zeaxanthin and lutein have been found at higher concentrations in a red-pigmented Italian landrace (6.6 and 2.2 μg/g DW, respectively) following by lower contents of β-cryptoxanthin and β-carotene (1.2 and 0.2 μg/g DW, respectively) (69).

Carotenoid concentrations observed in the current research are more comparable to those found in white maize, but lower than values reported in yellow and red-pigmented maize. Ryu et al. (70) pointed out that hard maize types such as pop, dent, and flint have significantly higher carotenoid contents than floury types. In addition, the endosperm fraction has been shown to contain the highest carotenoid concentrations in maize and in other cereal kernels, therefore yellow and orange endosperms have higher carotenoid concentrations than white endosperm (71, 72). *Cabanita* maize is an amylaceous floury type with white endosperms which likely explains its lower carotenoid levels than other maize landraces. Only sample COM showed a creamy-white endosperm among all samples and had the highest carotenoid values. The orange pigmentation of COM kernel pericarp could be partly linked to the presence of other not detected phenolic compounds in this study such as phlobaphenes. These flavan-4-ols polymers have been identified in Italian maize landraces with brick red pigmentation which is similar as that observed in COM sample (69). Phlobaphenes have been associated with resistance to mycotoxin-producing fungal infection in maize (73). Domínguez-Hernández et al. (16) have recently stated that the carotenoid characterization of maize landrace diversity is very limited and germplasm from few regions of the world including only Mexico and Brazil from the American continent have been investigated. Results from the present study contribute for the first time with information about the carotenoid composition of part of the maize diversity from Peru which is another important primary center of maize domestication in the world.

### *In vitro* antioxidant capacity

Only one sample from each location was selected based on its higher TPCs for the following *in vitro* antioxidant capacity assays. Table 6 shows the health-relevant antioxidant potential of the free and bound phenolic fractions from *Cabanita* maize samples using the DPPH and ABTS methods. In addition, the hydrophilic and lipophilic fractions were evaluated. The bound phenolic fractions from all *Cabanita* samples showed the highest DPPH and ABTS free radical scavenging capacity and represented 93.5–98.2% of the total antioxidant capacity (free + bound). A significant correlation was found between the UHPLC total bound phenolic contents and the antioxidant capacity (*r* = 0.6111 and *r* = 0.5675, *p* < 0.05 with the DPPH and ABTS methods, respectively). Bound hydroxycinnamic acids such as *p*-coumaric and ferulic acids were correlated with this property (*r* = 0.5168, *r* = 0.6058 and *r* = 0.5444, *r* = 0.5454, *p* < 0.05, with the DPPH and ABTS methods, respectively) suggesting a high contribution of these bound phenolic acids to the antioxidant potential of *Cabanita* maize. The free antioxidant capacity was highly correlated with the anthocyanin contents using both methods (*r* = 0.8367 and *r* = 0.8197, *p* < 0.05, DPPH and ABTS assays, respectively) indicating an important contribution of anthocyanins to the free antioxidant property in partially red and purple-pigmented kernels.

**TABLE 6:** *In vitro* antioxidant capacity of Cabanita maize samples from Caylloma and Castilla provinces.

Analyses	Caylloma					Castilla				
	CCR	COM	CAW	CLY	CHW	CHY	CAY	CSR	CPM	CALR
**DPPH Antioxidant assay (μ mol TE/100 g DW)**										
Free	82.4 ± 19.2a	38.8 ± 3.4ab	61.3 ± 3.9ab	34.6 ± 1.6b	42.2 ± 3.1ab	43.5 ± 8.6ab	58.1 ± 9.5ab	60.3 ± 3.0ab	93.2 ± 12.5a	56.4 ± 6.0ab
Bound	836.2 ± 30.4abc	641.9 ± 50.0d	673.9 ± 79.8cd	776.2 ± 33.8bcd	760.0 ± 51.5bcd	897.7 ± 61.9ab	947.7 ± 93.3a	827.7 ± 138.8abc	831.9 ± 23.6abc	900.6 ± 36.3ab
Total	918.6 ± 36.4ab	680.7 ± 48.7d	735.1 ± 79.8cd	810.8 ± 33.7bcd	801.9 ± 53.8bcd	941.1 ± 68.4ab	1005.8 ± 92.0a	888.0 ± 135.9abc	925.1 ± 35.8ab	957.0 ± 36.8ab
Hydrophilic fraction	348.4 ± 54.1ab	336.2 ± 23.1ab	268.2 ± 50.8bc	294.5 ± 26.9bc	325.3 ± 30.0ab	228.2 ± 29.5c	397.8 ± 34.8a	309.6 ± 15.3abc	290.6 ± 13.6bc	265.9 ± 31.4bc
Lipophilic fraction	35.4 ± 3.4ab	34.0 ± 3.9ab	24.1 ± 6.2b	31.2 ± 6.4ab	34.6 ± 5.3ab	39.9 ± 1.1ab	40.3 ± 10.0a	35.5 ± 4.8ab	33.7 ± 2.7ab	32.9 ± 5.7ab
**ABTS Antioxidant assay (μ mol TE/100 g DW)**										
Free	103.2 ± 28.4a	46.0 ± 11.0b	40.7 ± 5.6b	36.8 ± 9.1b	53.3 ± 5.1b	43.7 ± 4.3b	62.9 ± 22.1b	56.5 ± 3.1b	106.8 ± 21.0a	49.0 ± 7.7b
Bound	3228.8 ± 135.0bcde	2582.8 ± 327.2f	2585.3 ± 228.2ef	2823.5 ± 95.9def	2951.3 ± 237.8cdef	3801.5 ± 321.0ab	4040.7 ± 283.6a	3519.3 ± 379.8abc	3303.8 ± 206.0bcd	3810.0 ± 176.9ab
Total	3331.9 ± 156.6bcd	2628.8 ± 323.3e	2626.0 ± 224.2e	2860.3 ± 90.1de	3004.6 ± 238.4cde	3845.1 ± 316.9ab	4103.6 ± 280.4a	3575.8 ± 382.9abc	3410.6 ± 220.8bcd	3859.0 ± 182.5ab
Hydrophilic fraction	651.5 ± 41.6b	685.4 ± 23.2b	566.3 ± 11.1b	617.7 ± 48.3b	676.6 ± 32.0b	683.6 ± 50.7b	1068.9 ± 90.1a	689.4 ± 59.3b	652.0 ± 37.8b	661.9 ± 57.8b
Lipophilic fraction	85.0 ± 5.5ab	86.3 ± 3.1ab	73.5 ± 6.3abc	75.9 ± 5.2abc	82.3 ± 5.1ab	76.7 ± 8.0abc	87.3 ± 5.9a	63.1 ± 4.6c	72.0 ± 5.6bc	77.7 ± 7.5abc

The total ABTS antioxidant capacity (free + bound) from this research (2626.0–4103.6 μmol TE/100 g DW) is almost 1.4 times higher than levels reported by Ranilla et al. (24) in some *Cabanita* maize samples (1879.3–2942.1 μmol TE/100 g DW). This may be related to the higher phenolic contents found in this study as was previously discussed. Lower total antioxidant values than those from current investigation were determined in Chilean (1307–1850 μmol TE/100 g DW, ABTS method) and Southern Mexican maize landraces with different kernel pigmentations (377–484 μmol TE/100 g DW, DPPH method) (47, 64). In addition, comparable total antioxidant capacity has been observed in landraces from the Northeast of Mexico (2827–4264 μmol TE/100 g DW, ABTS method) and in diverse waxy maize genotypes grown in Thailand (average of 1096 and 3791 μmol TE/100 g DW with the DPPH and ABTS methods, respectively) (74, 75).

The hydrophilic antioxidant capacity (228.2–397.8 and 566.3–1068 μmol TE/100 g DW, with DPPH and ABTS assays, respectively) was higher than lipophilic antioxidant values (24.1–40.3 and 63.1–87.3 μmol TE/100 g DW, with DPPH and ABTS assays, respectively) which may be due to the higher phenolic concentrations found in *Cabanita* maize than lipophilic phytochemicals such as carotenoids. Significant positive correlations between the total free UHPLC phenolic contents and the hydrophilic antioxidant capacity (*r* = 0.6492, *r* = 0.7820, *p* < 0.05, DPPH and ABTS methods, respectively) were obtained. In case of the lipophilic antioxidant capacity, results showed moderate correlation with the total carotenoid levels (*r* = 0.4443, *p* < 0.05, ABTS method). Other minor lipophilic compounds not evaluated in this study may partially contribute to the lipophilic antioxidant property in *Cabanita* maize. Tocols and phytostherols have been also detected in maize (76). Recently, Lux et al. (50) found tocopherols and tocotrienols in similar ranges as those shown by total carotenoids in yellow maize.

*Cabanita* maize from Castilla showed higher total antioxidant capacity (free + bound) than Caylloma samples, and differences were high with the ABTS method. Ranges varied from 3410.6 to 4103.6 μmol TE/100 g DW and from 2626.0 to 3331.9 μmol TE/100 g DW for Castilla and Caylloma maize samples, respectively. CAY and CCR samples showed the highest total antioxidant capacity among Castilla and Caylloma maize, respectively. The hydrophilic and lipophilic antioxidant capacity results were in general similar in both provinces, and CAY sample (Castilla) showed the highest values (1068.9 and 87.3 μmol TE/100 g DW for the hydrophilic and lipophilic antioxidant capacity, ABTS assay). Current hydrophilic antioxidant capacity results are much higher than values reported in commercial Chinese maize (28 μmol TE/100 g DW, ABTS method), and other cereal grains such as sorghum (40 μmol TE/100 g DW, ABTS method), wheat (83 μmol TE/100 g DW, ABTS method), and barley (210–250 μmol TE/100 g DW, ABTS method) (77, 78). Comparable values have been shown in quinoa seeds (1280 and 22 μmol TE/100 g DW, hydrophilic and lipophilic fractions, respectively; ABTS method) (79). Differences may be attributed to the specific composition of hydrophilic and lipophilic phytochemicals associated with each grain food.

### Principal component analysis

**FIGURE 3:**
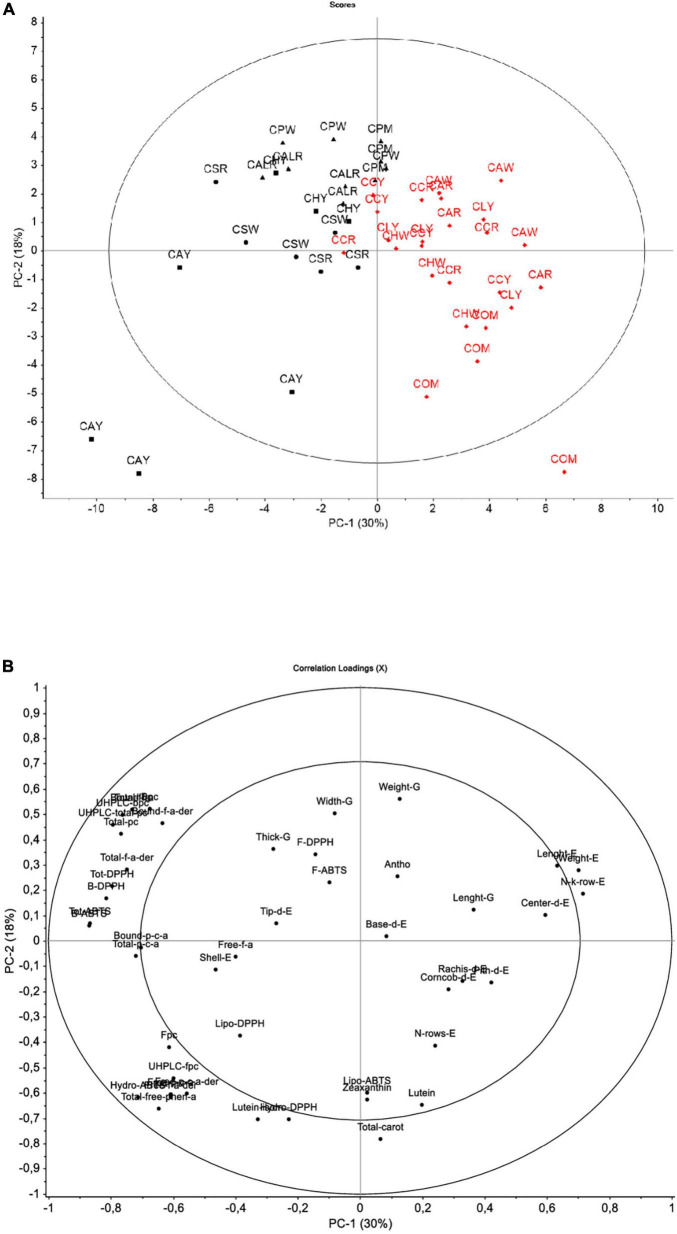
Score plot **(A)** and loading plot **(B)** for the principal component analysis (PCA) model for the first two factors considering of all data. In **(A)** Districts: box (Andahua), dot (Ayo), triangle (Chachas), diamond (Cabanaconde). Provinces: black (Castilla), red (Caylloma).

Underlying relationships based on all studied variables were explored through the PCA descriptive model (Figure 3). The score plot is shown considering the sample code and the district of origin of *Cabanita* samples. This approach retained two principal components (PC1 and PC2) which expressed 48% of the total variance of the sample data set likely indicating a certain grade of homogeneity among all evaluated maize samples from both provinces. This degree of homogeneity could be explained by the endemism of *Cabanita* race. Higher variability with the first two PC (61–71%) was reported by Ranilla et al. (24) when evaluating different maize races from a germplasm bank and collected *in situ* from the Arequipa region in Peru. In the same study, a clear discrimination based on the phenolic composition and some *in vitro* functional properties was found among the *Kculli* (purple-pigmented kernel), *Granada* (red-pigmented kernels), *Arequipeño* (white-yellow kernels) and *Cabanita* maize races (mostly white-pigmented kernels) (24).

Despite the observed low retained variability of the model, PC1 (30% of explained variability) separated samples from the Castilla (Chachas, Ayo, Andahua districts) and Caylloma (Cabanaconde district) provinces (from left to right, Figure 3A). The PC2 (18% of explained variability) separated maize grown in Chachas district (samples CALR, CPW, CPM) within the Castilla province. Differences between both provinces were increased by samples CAY (Andahua district, Castilla province) and COM (Cabanaconde district, Caylloma province). A positive correlation among the phenolic compounds including the bound and total UHPLC phenolic contents, bound ferulic acid, bound ferulic acid derivatives, total bound ferulic acid, bound and total *p*-coumaric acid was observed. These variables were correlated with the bound and total antioxidant capacity measured with the ABTS and DPPH free radical inhibition methods. All these variables were higher in samples from the Castilla province, specially from the districts of Chachas (CALR, CAPW, CPM), Ayo (CSW, CSR), and Andahua (CHY). CAY sample (Andahua district, Castilla) showed a different pattern among the other maize samples from Castilla (bottom left side of the score plot). This sample that showed higher TPCs as previously highlighted, also had the highest free UHPLC phenolic compounds including free ferulic acid derivatives, free *p*-coumaric acid derivatives and total free phenolic acids. These variables had a direct relation with higher ABTS hydrophilic antioxidant capacity in CAY sample (Figure 3B).

The loading plot also shows that yield-relevant physical characteristics were inversely correlated with phenolic concentrations and the *in vitro* antioxidant capacity indicating that Caylloma samples (right side of the score plot) are linked to lower phenolic contents but had better agronomic yield. Data from sample COM grouped differently compared with the rest of Caylloma maize (bottom right side of the score plot, Figure 3A). Variables such as lutein, and total carotenoid concentrations were the highest in this sample and were associated with high ABTS lipophilic antioxidant capacity. Further PCA analysis excluding data from CAY and COM samples was performed and results are shown in Supplementary Figure 5. The explained variability with the first two PC decreased to 42% indicating higher homogeneity among evaluated maize samples based on studied variables. However, PC1 (29% of explained variability) consistently separated data according to the province of origin (Castilla and Caylloma samples were in the left and right side of the score plot, respectively, Supplementary Figure 5A). Samples from Castilla continued to be linked to higher phenolic contents. Overall, most of the physical kernel and ear parameters (excepting those linked to the yield), and the carotenoid contents were not significant variables (Supplementary Figure 5B).

Several studies have emphasized that the genotype has more influence on the bioactive composition of maize than differences in the agroecological factors. Giordano et al. (80) found no differences in the main phenolic acids, anthocyanins, carotenoids (β-cryptoxanthin and β-carotene) and the antioxidant capacity from several pigmented Italian landraces under different nitrogen rates. However, the genotype showed a significant effect in same study (80). Some free and bound phenolic compounds along with lipophilic compounds such as carotenoids and tocochromanols from yellow maize were increased by sowing time, but not affected by phosphate fertilization (50). Furthermore, Paulsmeyer et al. (54) pointed out that the anthocyanin profiles and concentrations were strongly influenced by genetic factors with minimal influence of environmental conditions. Based on the PCA analysis in current study (low retained variability of the models), evaluated maize from Caylloma and Castilla provinces would belong to the same landrace population or race (*Cabanita*); however, the heterogenous Andean climatic factors seem to affect the bioactive composition of *Cabanita* maize to a higher extent than the sample type. Differences between maize from both provinces could be attributed to different agroecological factors. Districts from Castilla provinces showed more extreme climatic conditions than Caylloma locations (Supplementary Figures 1–4). The phenylpropanoid metabolism in plants has shown an extreme plasticity under changes in the environmental conditions redirecting the metabolic flux to produce phenolic-derived metabolites for plant protection (81). Increased levels of TPCs, bound ferulic acid, and DPPH antioxidant capacity have been reported in kernels from Peruvian purple maize grown at highland Andean locations (with lower temperature ranges and high UV radiation) compared with maize from lowland sites (18). The biosynthesis of ferulic acid and its derivatives was induced in maize seedlings and roots, stems, and leaves of maize plants under salt stress (82). The increase of phenolic metabolites under several abiotic stress factors has been previously reported in other cereal crops (83, 84). Cold stress has also shown to impair the photosynthesis leading to a decrease in grain yield (85). This may explain the lower yield physical parameters in maize from Castilla. Nevertheless, specific agricultural management differences observed in each province as previously stated could also play a role and should be investigated in future studies.

## Conclusion

The diversity of the Peruvian Andean maize race *Cabanita* from two provinces (Caylloma and Castilla) in the Arequipa region is a promising source of phenolic compounds with *in vitro* antioxidant capacity. Major free phenolic compounds in all maize samples were *p*-coumaric and ferulic acid derivatives whereas anthocyanins were only detected in samples with partially red and purple-pigmented kernels (CCR, CAR, CHW, CHY, CSR, CPM, CALR). The bound phenolic fractions were rich in ferulic acid and its derivatives, followed by *p*-coumaric acid. The hydrophilic antioxidant capacity was correlated with the free phenolic fraction, whereas bound phenolic acids highly contributed to the bound antioxidant capacity. Orange (COM) and white-yellow pigmented maize (CCY, CLY, CHW, CAY, CPW) showed carotenoid compounds mostly xanthophylls such as lutein and zeaxanthin. The multivariate analysis (PCA) revealed a low variability of integrated data indicating a grade of similarity among evaluated maize samples based on their physical, phytochemical, and antioxidant properties. However, Caylloma samples were characterized by their more uniform physical characteristics and higher yield than Castilla maize which exhibited higher phenolic contents and antioxidant capacity. Samples CAY (Castilla) and COM (Caylloma) were remarkable due to their highest phenolic and carotenoid concentrations among all samples (246.7 mg/100 g DW and 1.95 μg/g DW, respectively). The heterogeneous environmental conditions in the Andean region along with differences in the pre-harvest agricultural practices may play a role, but genetic factors may also be involved and should be further investigated. This research provides the foundations of metabolomic base for future molecular studies to better characterize the ethnic-relevant maize race *Cabanita*. Results from this research contribute to current efforts to ensure a good characterization of the high Peruvian maize diversity to give extra value to Peruvian biodiversity for the development of Andean indigenous health-targeted food systems.

## Data availability statement

The original contributions presented in this study are included in the article and Supplementary material. Further inquiries can be directed to the corresponding authors.

## Author contributions

LR and GZ conceived and designed the study. LR directed the research and wrote the manuscript. IF-C and RC-P performed the experiments and analyzed the data. SM-T helped with the experiments. HB-G coordinated and helped with the sample collection. CF performed the multivariate statistical analysis. GZ, CF, and KS critically reviewed the manuscript. All authors have read and approved the final manuscript.
