# Supplementary material for: Diversity of the Peruvian Andean maize (Zea mays L.) race Cabanita: Polyphenols, carotenoids, in vitro antioxidant capacity, and physical characteristics
Source: Front Nutr. 2022 Sep 26;9:983208. doi: 10.3389/fnut.2022.983208 (PMC9549777; doi:10.3389/fnut.2022.983208)
Supplement: Supplementary file 1 [file Data_Sheet_1.pdf]

Supplementary Material

1 Supplementary Figures

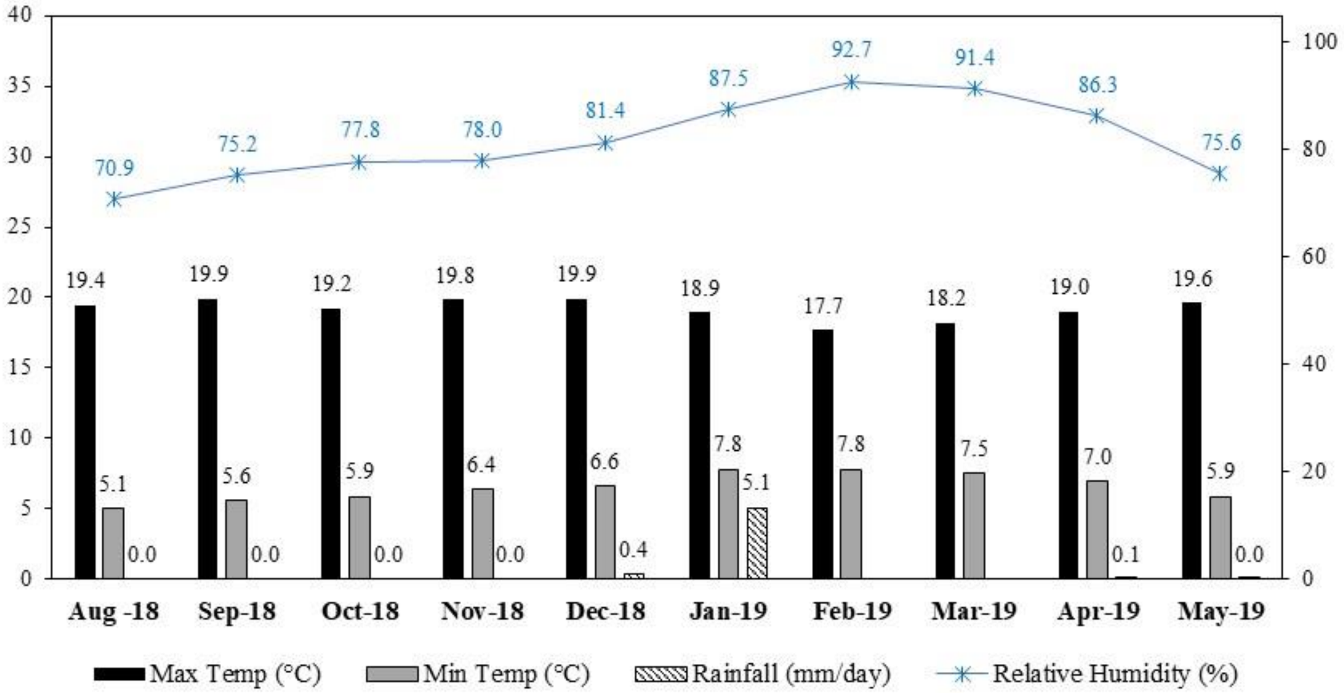

Supplementary Figure 1. Environmental conditions per month-year (values are the average per month of the maximum and minimum temperature, rainfall, and relative humidity) during the period of maize plants growth in the district of *Cabanaconde* (Caylloma province) (Adapted from data of the National Meteorology and Hydrology Service of Peru, SENAMHI) https://www.senamhi.gob.pe/main.php?dp=arequipa&p=estaciones)

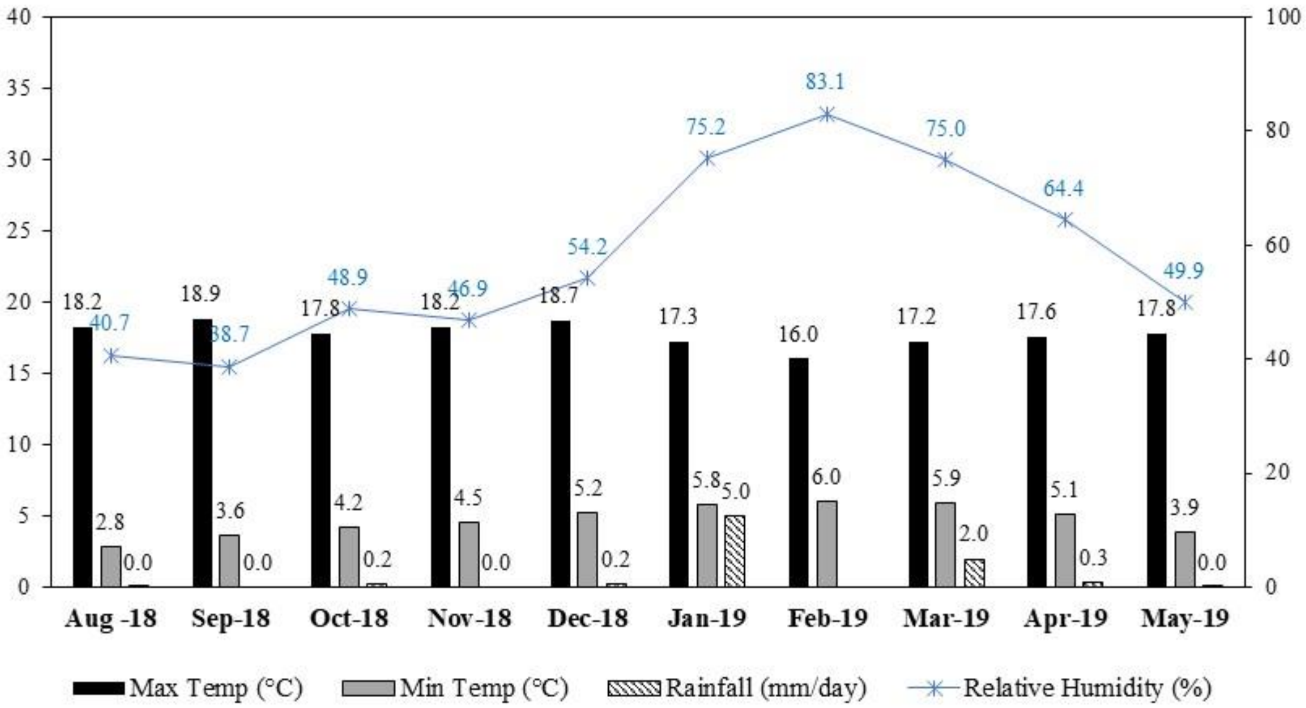

Supplementary Figure 2. Environmental conditions per month-year (values are the average per month of the maximum and minimum temperature, rainfall, and relative humidity) during the period of maize plants growth in the district of *Andahua* (Castilla province) (Adapted from data of the National Meteorology and Hydrology Service of Peru, SENAMHI) https://www.senamhi.gob.pe/main.php?dp=arequipa&p=estaciones)

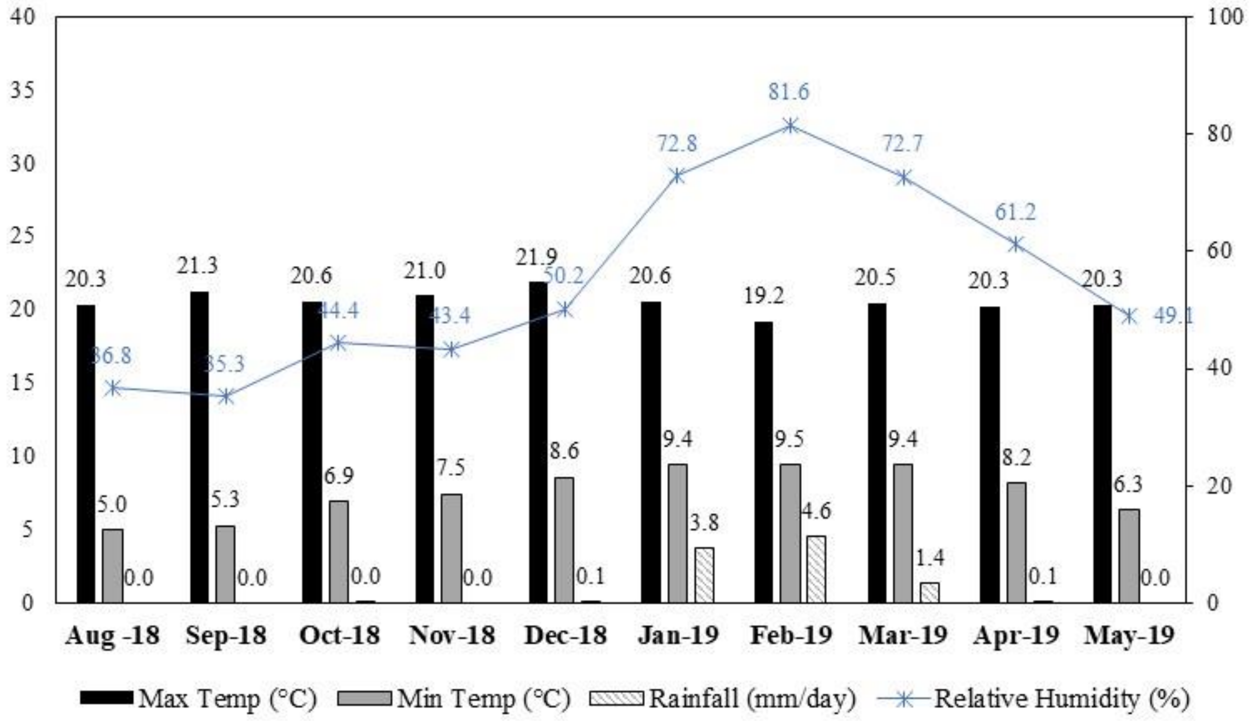

Supplementary Figure 3. Environmental conditions per month-year (values are the average per month of the maximum and minimum temperature, rainfall, and relative humidity) during the period of maize plants growth in the district of *Chachas* (Castilla province) (Adapted from data of the National Meteorology and Hydrology Service of Peru, SENAMHI) https://www.senamhi.gob.pe/main.php?dp=arequipa&p=estaciones)

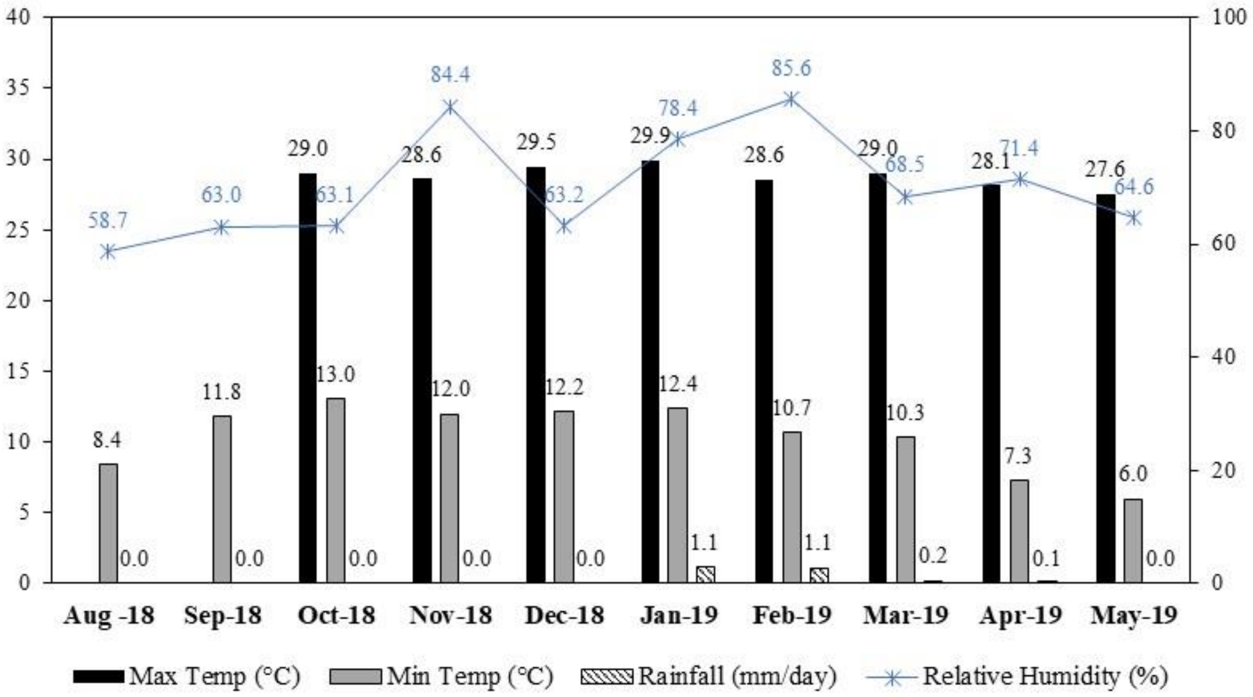

Supplementary Figure 4. Environmental conditions per month-year (values are the average per month of the maximum and minimum temperature, rainfall, and relative humidity) during the period of maize plants growth in the district of *Ayo* (Castilla province) (Adapted from data of the National Meteorology and Hydrology Service of Peru, SENAMHI) https://www.senamhi.gob.pe/main.php?dp=arequipa&p=estaciones)

A)

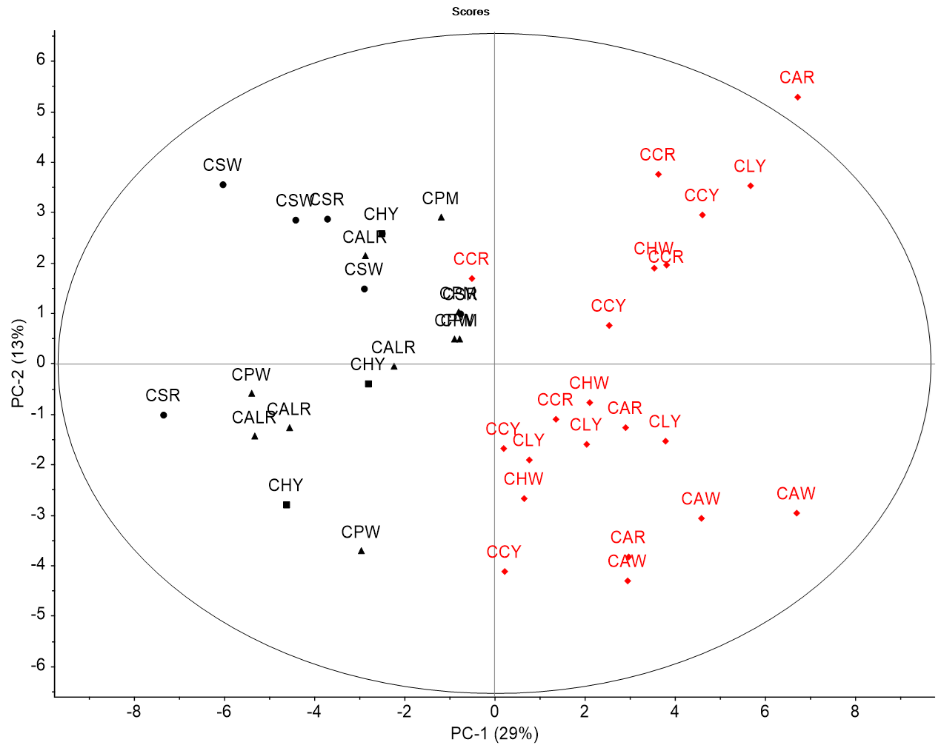

B)

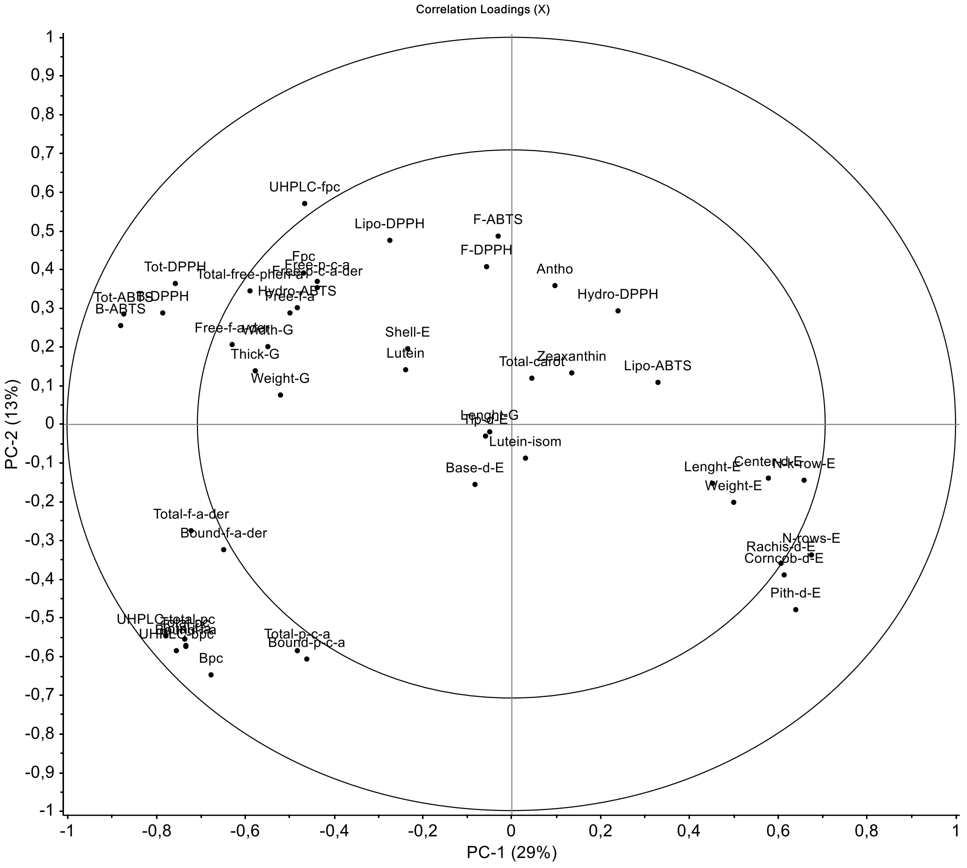

Supplementary Figure 5. Score plot (A) and loading plot (B) for the principal component analysis (PCA) model for the first two factors excluding data from CAY (Castilla) and COM (Caylloma) samples. In A; Districts: box (Andahua), dot (Ayo), triangle (Chachas), diamond (Cabanaconde). Provinces: black (Castilla), red (Caylloma)

2 Supplementary Tables

Supplementary Table 1. Pre-harvest agricultural management used by farmers from the Caylloma province for the cultivation of Cabanita maize samples

**Location**		**Auqui**	**Cusqui**	**Huancce -Tranca**	**Occollina - Tuntuiguita**	**Liguay**
**Code**		**CAW - CAR**	**CCR - CCY**	**CHW**	**COM**	**CLY**
**Seed**	**Pre-Treatment**	Drying and selection	Drying and selection	Drying and selection	Drying and selection	Dry and selection
	**Origin**	Exchange among the farmers from *Comision de Usuarios La Campiña*	Exchange among the farmers from *Comision de Usuarios La Campiña*	Exchange among the farmers from *Comision de Usuarios La Campiña*	Exchange among the farmers from *Comision de Usuarios La Campiña*	Exchange among the farmers from *Comision de Usuarios La Campiña*
**Other crops**	**Other previous species in the same land**	No	No	No	No	No
	**Crops close to the land**	No	No	No	No	No
	**Other species during maize growth**	Only weeds	Only weeds	Only weeds	Only weeds	Only weeds
**Sowing**	**Fallow stage (months)**	4	4	3	4	4
	**Soil preparation**	Land cleaning, organic fertilization with manure, tillage (before and during sowing), earth at rest after the 1st tillage, surface irrigation. Hilling after sowing.	Land cleaning, organic fertilization with manure, tillage (before and during sowing), earth at rest after the 1st tillage, surface irrigation. Hilling after sowing.	Organic fertilization with manure, compost, humus, and liquid organic fertilizer (*biol*), tillage (before and during sowing), earth at rest after the 1st tillage surface irrigation.	Tillage, earth at rest, organic fertilization with humus, surface irrigation.	Land cleaning, tillage, organic fertilization with manure, surface irrigation
	**Type**	Traditional plow with animals	Traditional plow with animals	Traditional plow with animals	Manual	Manual
	**Month-year**	September-2018	September-2018	August-2018	September-2018	September-2018
**Growth**	**Time (months)**	8	8	9	8	8
	**Use of herbicides**	Sometimes	Sometimes	No	No	No
	**Use of fertilizers**	No	No	Liquid organic fertilizer (*biol*) and compost	*Biol* Sometimes	No
**Presence of weeds**	**During growth**	*Briza spp.* (*tembladera*) *Medicago spp.* (*trebol carretilla*)	*Briza spp.* (*tembladera*) *Medicago spp.* (*trebol carretilla*)	*Briza spp.* (*tembladera*) *Medicago spp.* (*trebol carretilla*)	*Medicago spp.* (*trebol carretilla*)	*Briza spp.* (*tembladera*) *Medicago spp.* (*trebol carretilla*).
	**Control**	Manual	Manual	Manual	Manual	Manual
**Presence of pests**	**During growth**	*Spodoptera spp. (gusano cogollero)*	*Spodoptera spp.* (*gusano cogollero*)	*Spodoptera spp. (gusano cogollero), Sitophilus spp.* (*gorgojo*)	*Spodoptera spp. (gusano cogollero)*	*Spodoptera spp. (gusano cogollero)*
	**Control**	Flood irrigation	Flood irrigation	Organic repellents (based on wild *Lupinus mutabilis*, and *Capsicum pubescens*)	Flood irrigation	Flood irrigation
**Irrigation**	**Source**	Local water channel (*Majes*)	Local water channel (*Majes*)	Local water channel (*Majes*)	Local water channel (*Majes*)	Local water channel (*Majes*)
	**Type**	Gravity irrigation	Gravity irrigation	Gravity irrigation	Gravity irrigation	Gravity irrigation
**Harvest**	**Month-year**	May-2019	May-2019	May-2019	May-2019	May-2019
	**Type**	Manual	Manual	Manual	Manual	Manual

Supplementary Table 2. Pre-harvest agricultural management used by farmers from the Castilla province for the cultivation of Cabanita maize samples

**District**		**Andahua**		**Ayo**	**Chachas**	
**Location**		**Huancarani**	**Ajocha**	**Subna**	**Alleachaya**	**Pulluguaya**
**Code**		**CHY**	**CAY**	**CSW – CSR**	**CALR**	**CPW - CPM**
**Seed**	**Pre-Treatment**	Drying and selection	Drying and selection	Drying and selection	Drying and selection	Drying and selection
	**Origin**	Exchange among the farmers from the surrounding localities in the Castilla province	-	Exchange among the farmers from the surrounding localities in the Castilla province	Exchange among the farmers from the surrounding localities in the Castilla province	-
**Other crops**	**Other previous species in the same land**	Yes. *Ullucus tuberosus, Solanum tuberosum, Medicago sativa, Chenopodium quinoa, Pisum sativum*	Yes. *Ullucus tuberosus, Medicago sativa, Chenopodium quinoa*	Yes. *Solanum tuberosum, Vicia faba*	Yes. *Hordeum vulgare, Avena sativa*	Yes. *Pisum sativum, Vicia faba, Solanum tuberosum*
	**Crops close to the land**	No	No	No	No	No
	**Other species during the maize growth**	No	Yes. *Vicia faba* (habas)	Yes. *Medicago sativa*	Yes. *Medicago sativa*	No
**Sowing**	**Fallow stage (months)**	4	4	4	3	4
	**Soil preparation**	Land cleaning with cattle, organic fertilization with manure, tillage, earth at rest	Land cleaning with cattle, organic fertilization with manure, tillage, earth at rest	Land cleaning, organic fertilization with manure, tillage.	Land cleaning, organic fertilization with manure, tillage.	Land cleaning, organic fertilizati on with manure, tillage.
	**Type**	Traditional plow with animals	Traditional plow with animals	Traditional plow with animals	Traditional plow with animals	Traditional plow with animals
	**Month-year**	September-October-2018	September-October-2018	September-2018	September-October-2018	September-October-2018
**Growth**	**Time (months)**	8	8	8	8	8
	**Use of herbicides**	-	No	-	-	-
	**Use of fertilizers**	Manure	Manure	Manure	Manure	Manure
**Presence of weeds**	**During growth**	-	*Briza spp.* (*tembladera*)	-	-	-
	**Control**	Manual	Manual	Manual	Manual	Tillage, hoe
**Presence of pests**	**During growth**	*Helicoverpa spp. (gusano mazorquero)*	*Helicoverpa spp. (gusano mazorquero)*	*Helicoverpa spp. (gusano mazorquero)*	*Helicoverpa spp. (gusano mazorquero)*	*Spodoptera spp. (gusano cogollero)* Fungi
	**Control**	Flood irrigation	Flood irrigation	Manual and ash application	Seed treatment with insecticides	No control
**Irrigation**	**Source**	Local water channel (Andahua)	Local water channel (Andahua)	Local water channel (Andahua)	Local water channel (Andahua)	Local water channel (Andahua)
	**Type**	Gravity irrigation	Gravity irrigation	Gravity irrigation	Gravity irrigation	Gravity irrigation
**Harvest**	**Month-year**	June-2019	June-2019	June-2019	May-2019	June-2019
	**Type**	Manual	Manual	Manual	Manual	Manual
